# Necrosis of the Ribs, Complicating Pott’s Disease

**Published:** 1891-08

**Authors:** Louis A. Weigel

**Affiliations:** Rochester, N. Y.; Orthopedic Surgeon to the Free Out-Patient Department of the Rochester City Hospital; 54 N. Clinton Street


### NECROSIS OF THE RIBS, COMPLICATING POTT'S DISEASE.

By LOUIS A. WEIGEL, M. D., Rochester, N. Y.
Orthopedic Surgeon to the Free Out-Patient Department of the Rochester City Hospital.

Necrosis of the ribs, as a complication of Pott's disease, is sufficiently rare to merit record. The following case is of interest, as an illustration of this unusual complication:

Rebecca R., colored, *æt.* twenty-eight years, gives a history of an injury dating back about nine years, when she fell on the ice and wrenched her back. Previous to the injury, she states, her back was always weak, and she had a lateral curvature of the spine. Some time after the injury, a kyphotic prominence of the seventh and eighth dorsal spines showed itself, which was treated with plaster-of-Paris jackets for about two years. While under treatment her general health improved, and she was more free from pain. Soon after discontinuing the jacket treatment, an abscess appeared in the lumbar region; but after repeated aspiration she finally recovered from this. She now remained in fair health for about five years; during this time, however, the antero-posterior curvature gradually increased. In January, 1889, an abscess appeared in the left dorsal chest, just below the scapula, which was opened. On May 11, 1889, she was admitted to the Rochester City Hospital as a medical patient. At that time she had a distressing cough, profuse purulent expectoration, night sweats, etc. The dorsal abscess was discharging very profusely, and she suffered considerable pain in the back. Under treatment she improved in general health, but the condition of her back remained unchanged.

She came under my care in July, 1889. As she was unwilling to remain in the hospital, she was made an out-patient in August, 1889. The abscess was treated by irrigation and iodoform tents. The discharge of pus, however, continued to be very profuse, and she again began to fail in health. As it was evident that the pus

1. Read before the Medical Society of the State of New York, Feb. 3, 1891.

proceeded from diseased bone, which was readily detected by probing, she was induced to re-enter the hospital for the purpose of operative interference, October 21, 1890. Operation October 24, 1890. An incision, about five inches long, was made, extending nearly horizontal from the orifice of the old sinus. With a cross incision the ribs were fully exposed, and the eighth rib found to be extensively diseased. The articular extremity of this rib was nearly detached from the vertebra, the articulation being completely disorganized by the necrotic process. Upon examining the surrounding structures it was found that the sixth and seventh ribs were also diseased. The lower border of these two ribs was serrated and undermined, the disease being more extensive on the anterior than on the posterior surface. To the left of, and parallel with, the spinal column, a sinus extended downward about four inches. This was, probably, the remains of the lumbar abscess. The necrotic portion of the eighth rib was carefully detached from the underlying structures and resected. The carious portions of the sixth and seventh ribs were removed with gouge forceps, a drainage-tube inserted, and an antiseptic dressing applied.

There was no marked reaction following the operation. There is nothing specially to be noted in the subsequent course of the case, except that the pulse remains comparatively rapid, which condition existed previous to the operation. The pulse fluctuates between 90 and 130. The temperature after the operation showed no regularity in its rise and fall. The highest evening temperature is recorded on the twelfth day after the operation, when it reached 102.6°. The respiration is accelerated, which might be expected in a patient whose thorax is greatly deformed and distorted.

Since the operation, the patient's general health has greatly improved, and she expresses herself as feeling better than she has for months before. Her pulse yesterday A. M. was 90 ; temperature, normal. For some time after the operation, the discharge of pus was quite profuse, but within the past month it has greatly diminished in quantity.

When abscesses occur in the course of Pott's disease, they usually follow the line of least resistance, and manifest themselves in the lumbar, iliac, or psoas regions. Even when the tubercular disease exists in the transverse processes, which lie posterior to the ribs, it is uncommon for an abscess to appear in the lateral dorsal region ; and hence infection of surrounding structures is not likely to occur.

In the case reported, the disease of the ribs was, undoubtedly, secondary to the caries of the dorsal vertebræ, due to an extension of the tubercular process by continuity of structure. This would be a reasonable assumption, at least, with reference to the eighth rib. But the carious condition of the fifth and sixth ribs, at a point quite remote from the seat of disease in the spine, cannot be explained in this way.

Opinions differ as to the advisability of operative interference with the abscess, and resection of the ribs. Thus Albert[1] says he has never seen the slightest favorable influence upon the carious process follow an operation, and believes that opening an abscess occasionally hastens a fatal result. With reference to resection, he admits that not a few cases of cure are recorded; but is inclined to think that the favorable ones only are published. He has operated but twice, and failed to get a recovery, or even an improvement in the general condition of the patient. Both of his patients subsequently died of phthisis.

Israel[2] reports a case, in which he resected a portion of the twelfth rib, and scraped out the carious portion of the diseased vertebral body, in a patient thirty-four years of age. The patient did well for five weeks, when an empyema resulted, and death followed.

Notwithstanding these unfavorable opinions, the justifiability of operative interference, in cases similar to the one reported, seems to me to be beyond question.

[Note.—The patient was discharged from the hospital, convalescent, April 29, 1891. The amount of pus discharged is very small, the patient has gained about fifteen pounds in weight, and is in excellent general condition.]

54 N. Clinton Street.

---

Asthma Powder.—

| | | |
|---|---|---|
| ℞—Pulv. stramonii fol., | 32 | grammes. |
| Pulv. belladonnæ fol., | 32 | " |
| Pulv. potass. nit., | 4 | " |
| Pulv. opii, | 1 | gramme. |

Burn a little and inhale the fumes.

1. Albert, Chirurgie, Bd. 2, p. 174.

2. Bradford & Lovett, Orthop. Surg., p. 96.

